# Integral Fixation Titanium/Polyetheretherketone Cages for Cervical Arthrodesis: Evolution of Cage Design and Early Radiological Outcomes and Fusion Rates

**DOI:** 10.1111/os.12413

**Published:** 2019-01-06

**Authors:** Kevin Phan, Matthew H Pelletier, Prashanth J Rao, Wen Jie Choy, William R Walsh, Ralph J Mobbs

**Affiliations:** ^1^ University of New South Wales, Prince of Wales Hospital Randwick New South Wales Australia; ^2^ Surgical and Orthopaedic Research Laboratories Prince of Wales Clinical School, Prince of Wales Hospital Randwick New South Wales Australia; ^3^ Neuro Spine Clinic Prince of Wales Hospital Randwick New South Wales Australia; ^4^ Department of Spine Surgery Prince of Wales Hospital Randwick New South Wales Australia; ^5^ NSURG Research Group Prince of Wales Private Hospital Sydney New South Wales Australia

**Keywords:** Anterior cervical discectomy and fusion, Composite titanium/PEEK, Integral fixation ACDF cage

## Abstract

**Objective:**

To evaluate the initial outcomes of a composite cage with integral fixation using the Redmond titanium (Ti)/polyetheretherketone (PEEK) anterior cervical discectomy and fusion (ACDF) device.

**Methods:**

Data from 50 consecutive patients were prospectively collected from a single senior surgeon cohort. All cages were between 5 and 8 mm in height, and were packed with supercritical CO_2_ sterilized allograft. Patients were followed up for a minimum of 6 months, and implant complications were assessed.

**Results:**

From the original cohort, three were unavailable for follow‐up. Forty‐seven patients with a total of 58 operative levels were observed for a mean of 7.9 months. A fusion rate of 96% was achieved. Good to excellent outcomes were seen in 92% of patients. There were no cases of implant Ti/PEEK delamination or implant failure, with excellent early fusion rates using supercritical CO_2_ allograft.

**Conclusions:**

The present study demonstrates the development of a composite ACDF cage design that is a safe and effective treatment option with the potential for early osseointegration and interbody fusion. Supercritical CO_2_ sterilized allograft was an effective graft material supporting fusion.

## Introduction

Since its description by Robinson and Smith in 1955, anterior cervical discectomy and fusion (ACDF) has been widely used for the treatment of symptomatic cervical spondylosis and disc herniation that is unresponsive to conservative management^1^. Although the use of autograft is considered the gold standard in achieving fusion, due to associated short‐term and long‐term complications of graft harvest, interbody cage implants with a variety of bone graft substitutes have become the mainstay of ACDF^2^.

Historically, three key materials have been used in the creation of cervical cages: titanium (Ti) and its alloys, polyetheretherketone (PEEK), and carbon fiber‐PEEK. Due to the synovitis and lymphatic spread of fiber debris associated with radiolucent carbon fiber‐PEEK cages, Ti and PEEK are preferred in current designs. Both materials have advantages and disadvantages^3^, ^4^, ^5^, ^6^. Although Ti has shown extensive ability to support osseointegration^7^, PEEK is radiolucent, allowing for easier fusion assessment and has an elastic modulus matched closer to bone, theoretically reducing levels of subsidence^8^. Hypothetically, the improved osteoconductivity of Ti can be utilized in combination with the elastic modulus and radioopacity of PEEK through the creation of composite Ti/PEEK spacers^9^, ^10^, ^11^. Clinically available composite spacers combine a PEEK body with Ti‐endplates to theoretically augment bone‐implant fusion; however, to the author's knowledge, there are only two clinical studies evaluating their usage^12^, ^13^.

**Figure 1:**
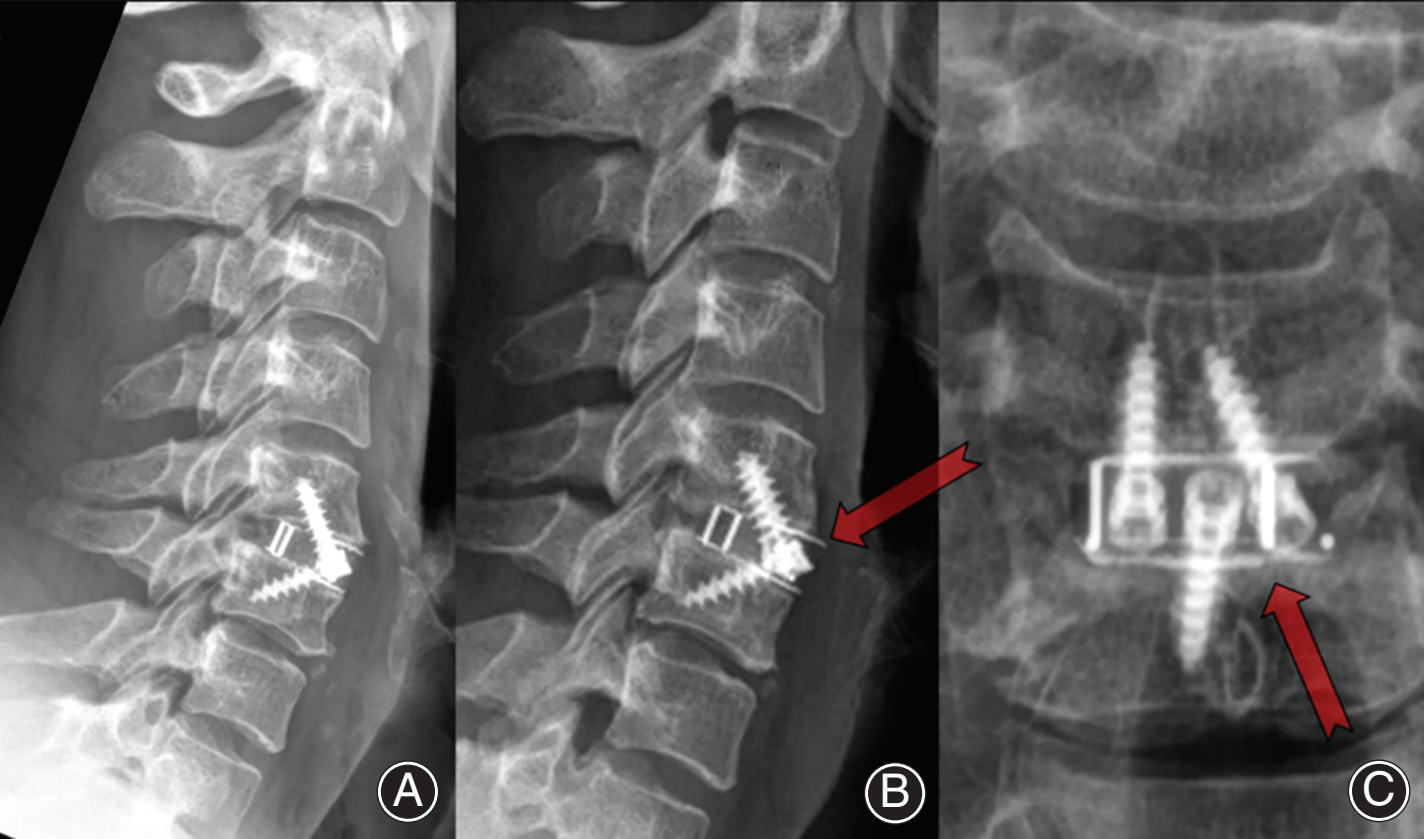
Potential issues with spray titanium (Ti)/polyetheretherketone (PEEK) cages. (A) Day 1 postoperation C_5/6_ anterior cervical discectomy and fusion (ACDF) using spray Ti/PEEK integral fixation cage; (B) 9 months postoperation with anterior cage migration (arrow). (C) Incongruous titanium spray surface with potential delamination.

There is little investigation in the literature into whether impaction of titanium‐coated PEEK cages into the disc space can result in wear or delamination of the coating. The senior author has observed 1 case of titanium wear with potential delamination (Fig. 1), which represents the first such reported case. Kienle *et al*.^14^ report that titanium cages with subtractive surface etching (no coating) are less susceptible to such delamination and failure.

The design modification of ACDF cages to include integral fixation *via* two or three screws has become increasingly popular due to the avoidance of anterior plate fixation that requires more extensive dissection and bone preparation. Recent evidence supports the use of these devices for anterior cervical fusion over anterior plate constructs^15^. The aim of the present study is to report early clinical and radiological outcomes of a unique design integral fixation Ti/PEEK cage.

## Materials and Methods

### *Ethics Approval*

Approval was obtained from the South Eastern Sydney Local Health District‐Northern Sector (SESLHD‐NS) ethics committee, Ref: HREC 11/183.

### *Patient Data*

Over a 6‐month period from July 2016 to December 2016, 50 patients were operated on and data was collected prospectively from a single senior surgeon cohort (RJM).

Inclusion criteria: (i) patients aged between 18 and 75 years; (ii) patients suffering from cervical traumatic or degenerative disease; and (iii) patients unsuitable for or unresponsive to conservative treatment.

Exclusion criteria: (i) patients suffering from significant comorbidities, including systemic infection and terminal cancer; (ii) patients with posterior longitudinal ligament ossification; and (iii) patients with osteopenia on preoperative bone mineral density were considered acceptable (however, patients with osteoporosis were not included in the current study).

**Figure 2:**
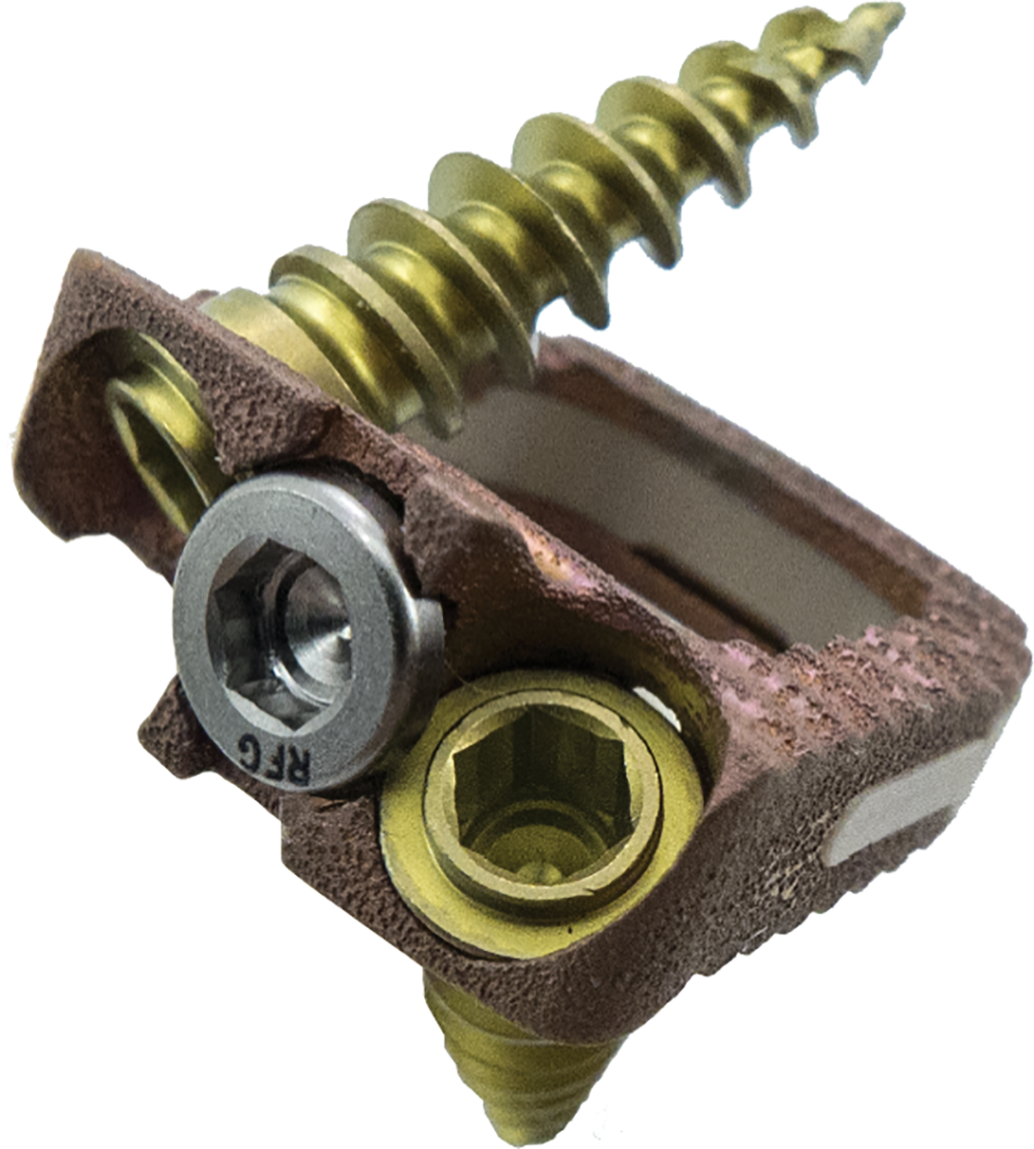
Redmond (A‐Spine, Asia, Taiwan, China), composite titanium (Ti)/polyetheretherketone (PEEK) integral fixation spacer featuring ridged titanium alloy endplates in combination with a PEEK body.

Patients were followed up for a minimum of 6 months, with assessments postoperatively at day 1, weeks 2 and 6, and months 3 and 6. All patients were operated on using the composite integral fixation Ti/PEEK Redmond cage (A‐Spine ASIA, Taiwan, China) (Fig. 2). This cage includes features of an integral fixation ×2 screw design, with porous Ti‐endplates, and a PEEK body and is available in the dimensions 14 mm × 15 mm (depth × width), 15 mm × 15 mm, 15 mm × 17 mm and 16 mm × 18.5 mm, ranging in height from 5 mm to 8 mm. Implant size choice was based on: (i) the height of the implant to mirror an adjacent normal disc height; and (ii) the maximum coverage of the endplate to distribute the load evenly.

### *Implant*

The integral fixation titanium/PEEK cage was constructed with a porous titanium plate architecture rather than a spray coating, to reduce the risk of delamination/wear of the implant surface. The integral fixation design consists of ×2 screws to prevent implant migration with an anterior anti‐backout plate (Fig. 2).

### *Indications*

**Figure 3:**
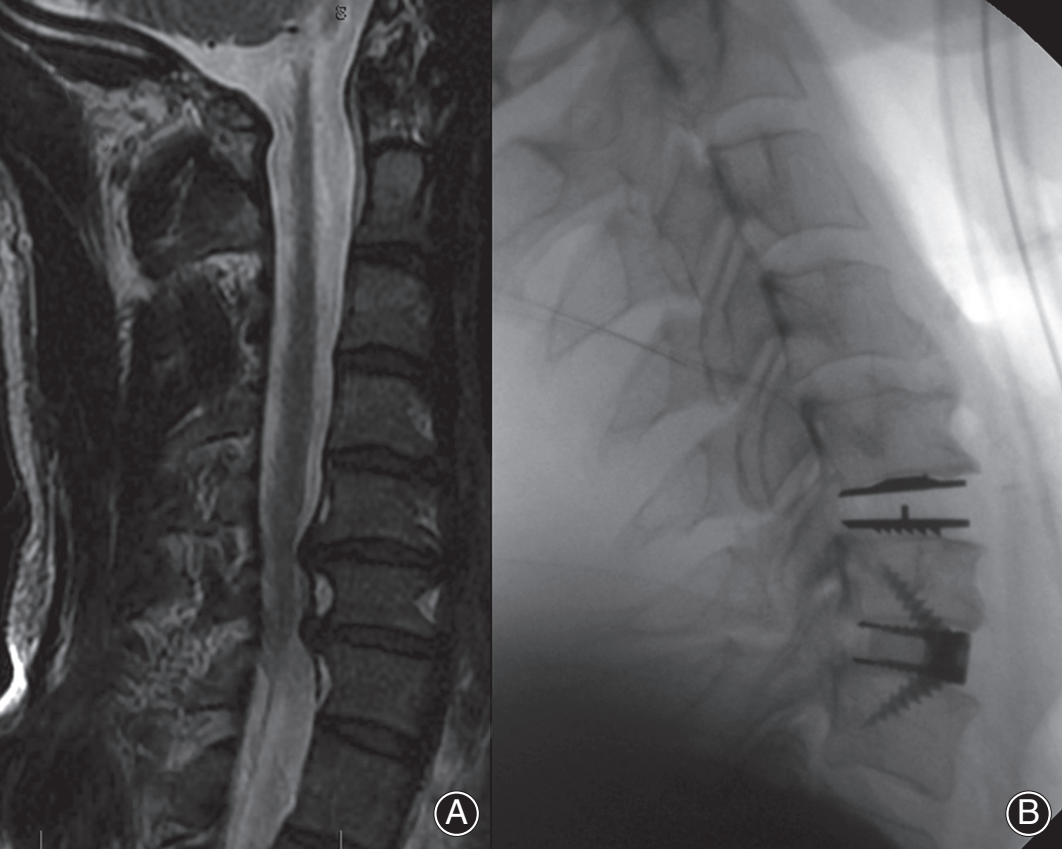
Anterior cervical discectomy and fusion (ACDF)/disc replacement hybrid for disc height loss and foraminal stenosis. (A) Saggital MRI of cervical level. (B) Intraoperative X‐ray.

**Figure 4:**
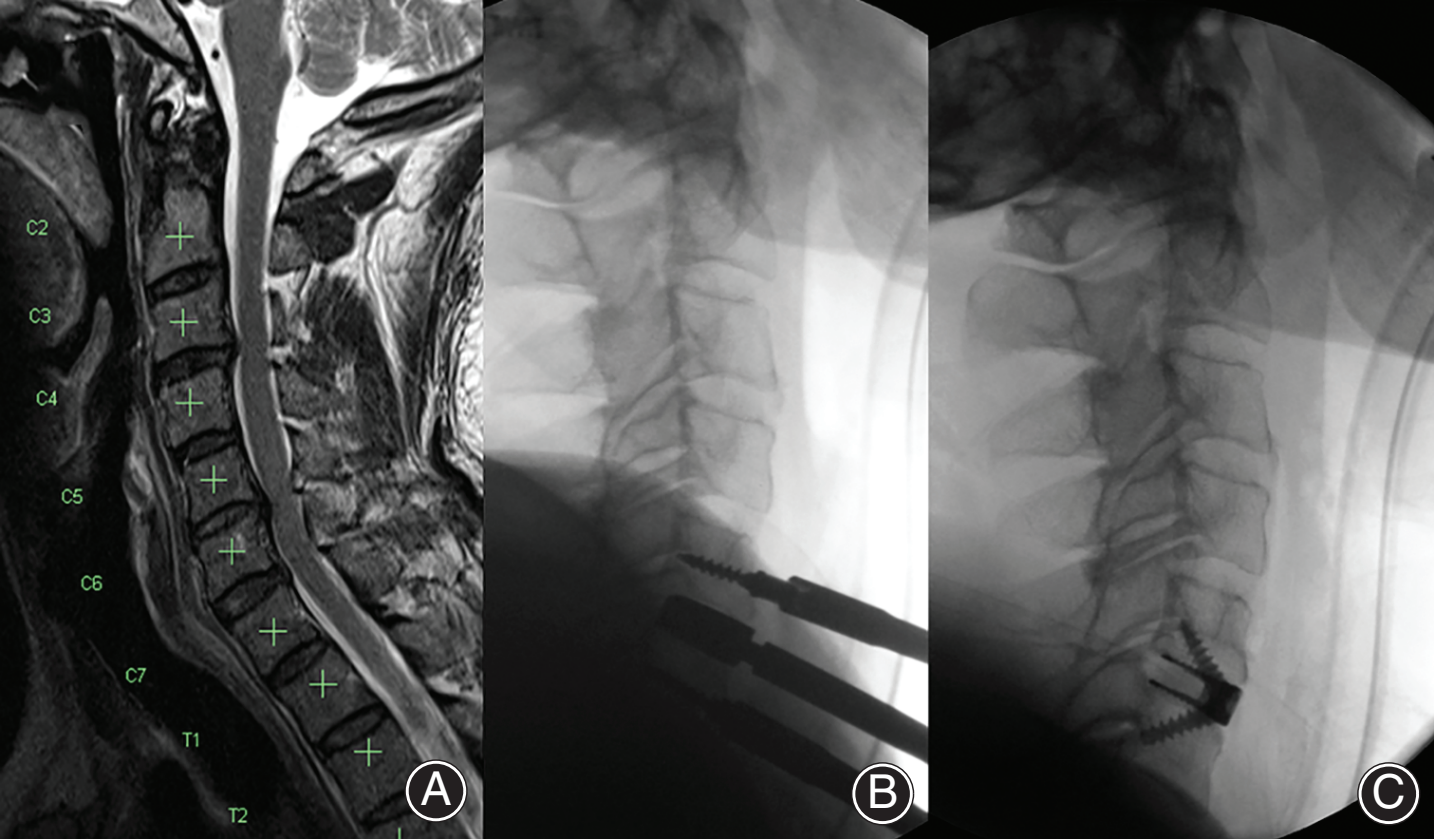
(A) Myelo/radiculopathy at C_5‐6_. (B) Trial prosthesis to check cage position and height. (C) Device in position.

**Figure 5:**
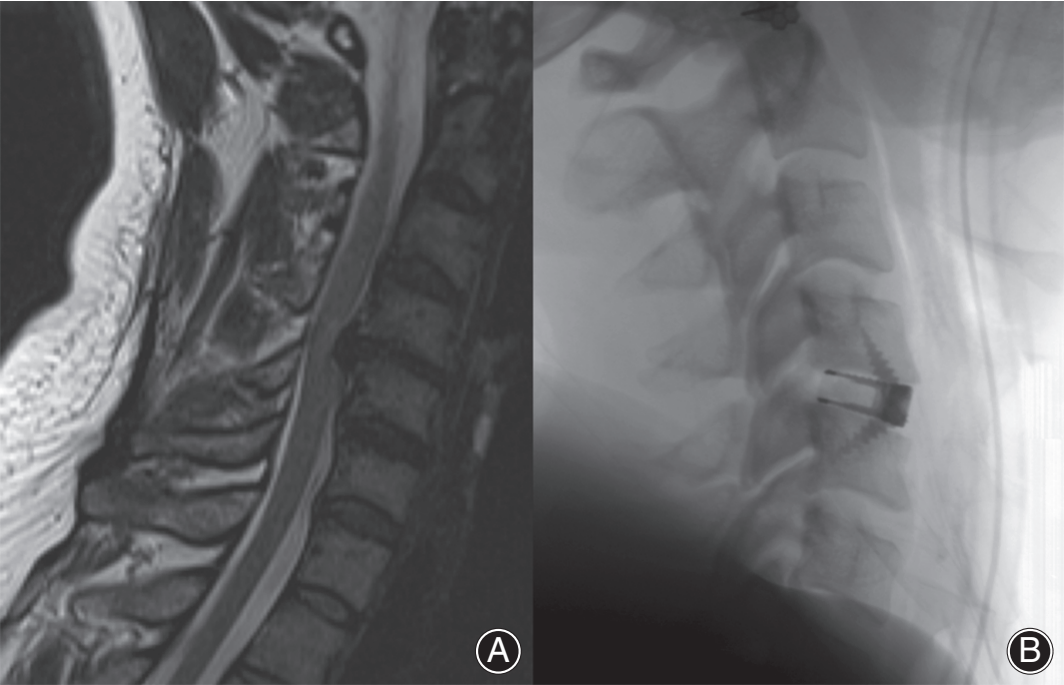
(A) Myelopathy at C_4‐5_ due to large central disc herniation. (B) Device in position.

**Figure 6:**
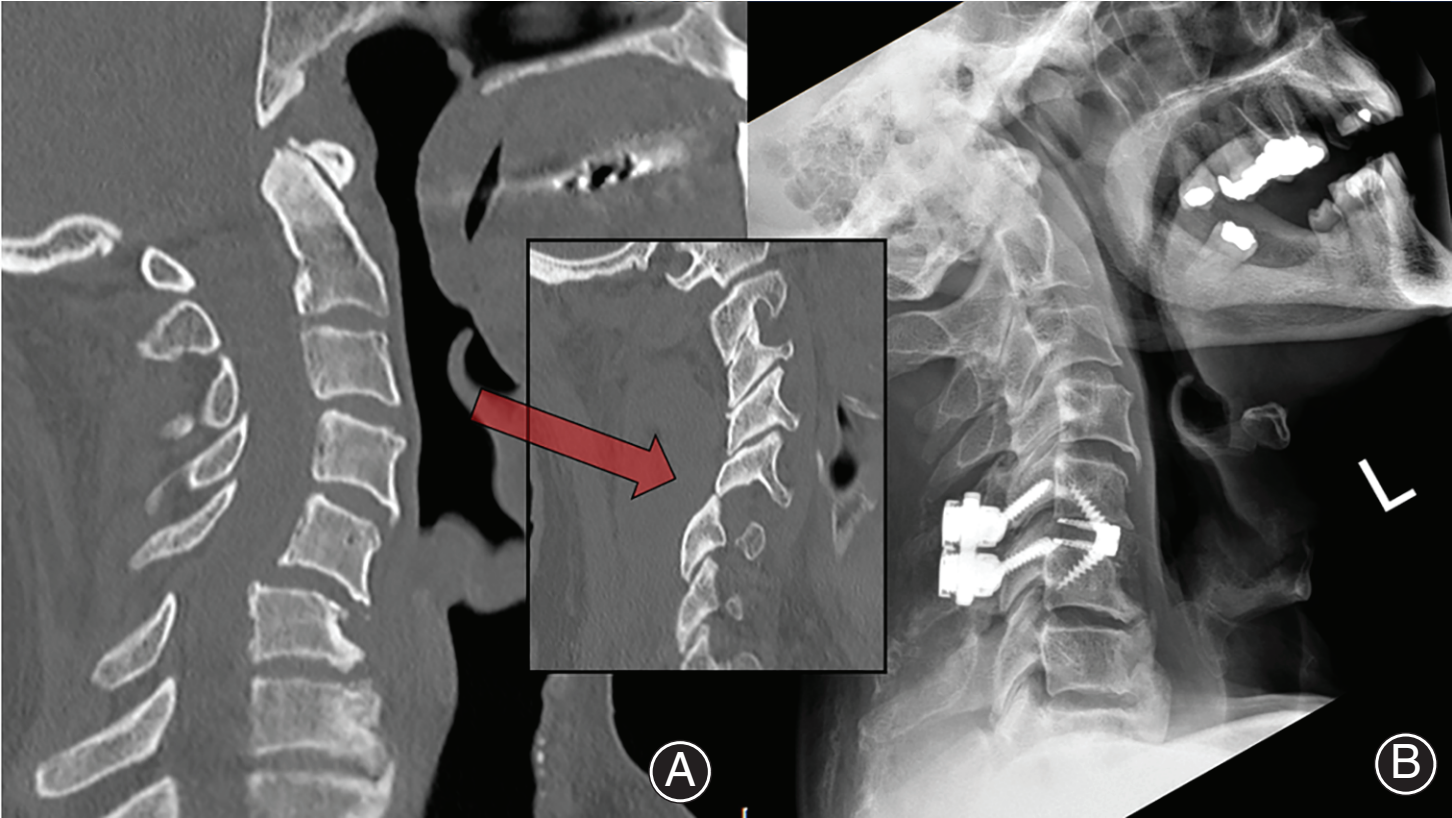
(A) Pre‐operative CT scan showing trauma with (inset) Bifacetal dislocation at C_5‐6_. (B) Anterior interbody fixation, with posterior lateral mass fixation (Neon 2 Posterior Cervical, Ulrich, Germany).

**Figure 7:**
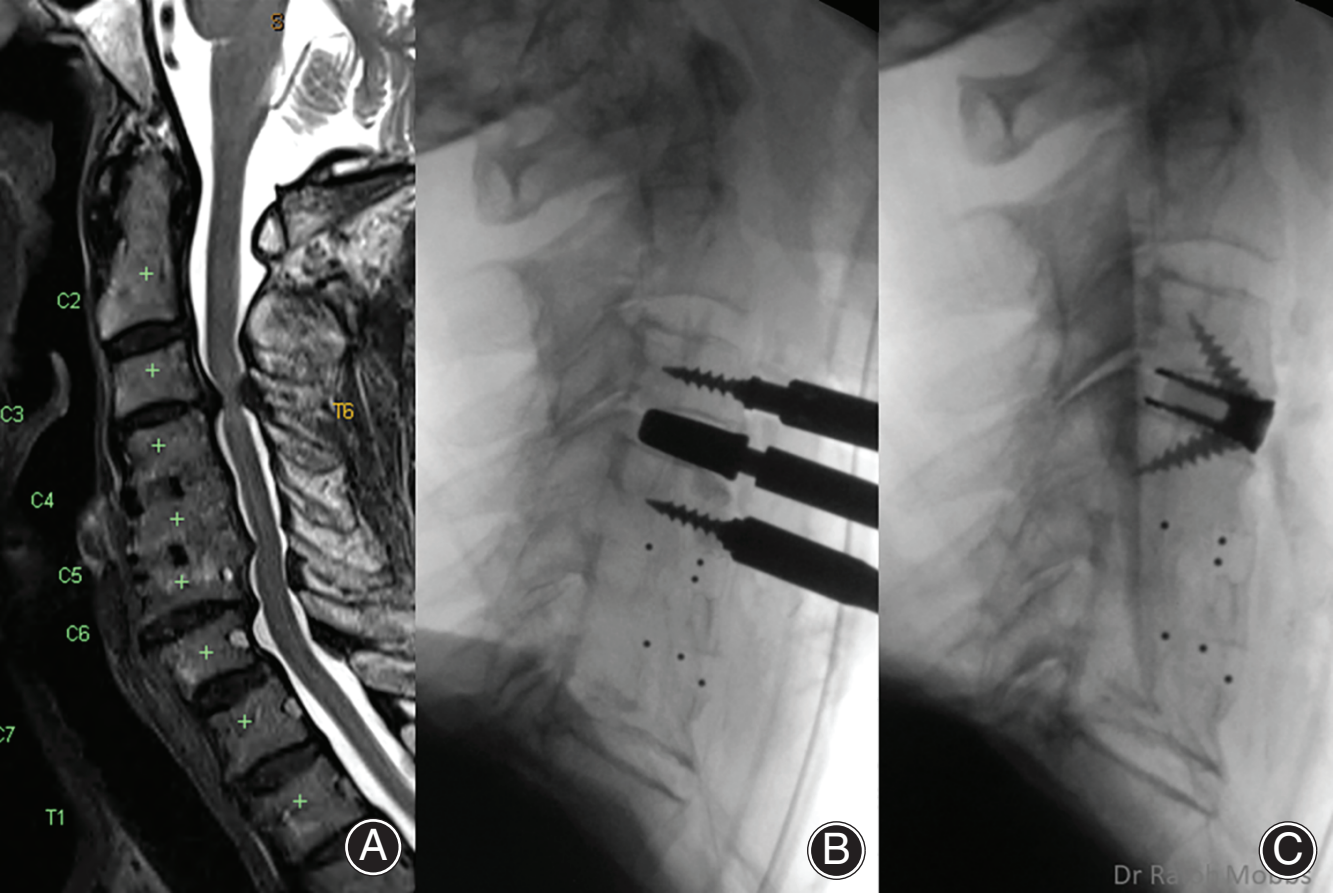
Adjacent segment degeneration: 83‐year‐old female with progressive myelopathy and a background of C_4‐5_ and C_5‐6_ ACDF performed 11 years prior. (A) Severe canal stenosis C_3‐4_ with cord signal change. (B) Intraoperative trial of prosthesis to confirm height and depth. (C) Final implant position.

The indications for ACDF in this patient cohort of 50 patients include 48 cases of degenerative pathologies and 2 cases of trauma. Examples of indications are included in Figs 3 and 4 (foraminal stenosis with radiculopathy/hybrid procedure), Fig. 5 (myelopathy) and Fig. 6 (trauma with bifacetal dislocation and spinal cord injury). Both trauma cases were bifacetal dislocation with neurological deficit, and operative management included a combination anterior and posterior decompression, realignment and fusion (Fig. 6). The degenerative pathologies included 29 patients with radiculopathy due to disc/osteophyte complex with foraminal stenosis (Fig. 3), 10 patients with myelopathy (Fig. 4), 6 patients with discogenic neck pain without radiculopathy, and 3 patients with adjacent segment degeneration with progressive neurological deterioration (Fig. 7). Clinical outcomes were based on the cohort of patients with degenerative pathologies only, with exclusion of the trauma cohort as both patients suffered an ASIA‐B neurological injury.

### *Surgical Technique*

All surgeries were conducted by a single senior surgeon using a modified Smith–Robinson technique under general anesthesia. Following a linear right anterolateral incision, retraction of the musculo‐visceral column was achieved using the Trim‐Line retractor system with distraction of the vertebral bodies using modified Caspar retraction pins (A‐Spine ASIA, Taiwan, China). Pathological disc material was then removed using a combination of rongeurs and curettes under the direct observation of an operating microscope (Pentero, C. Zeiss, Germany). Any visible osteophytes were also removed using a high speed 2‐mm drill and the posterior longitudinal ligament was divided and removed in all cases. Complete decompression and visualization of the dura and nerve roots was achieved. Decortication of the vertebral endplates was performed to optimize the bone‐cage/graft interface.

The appropriate size cage was determined through the usage of a trial spacer to confirm the height of the disc space (Fig. 3B). Cages were filled with human allograft (Allovance Cortico‐Cancellous Crunch (1–3.5 mm), Australian Biotechnologies, Sydney, Australia) that was processed and sterilized using a proprietary supercritical carbon dioxide process. The allograft was firmly packed into the cage with the aim of distributing the axial loading through the implant. Cages were inserted using standard instrumentation and tapped into place (Fig. 3A–C).

**Figure 8:**
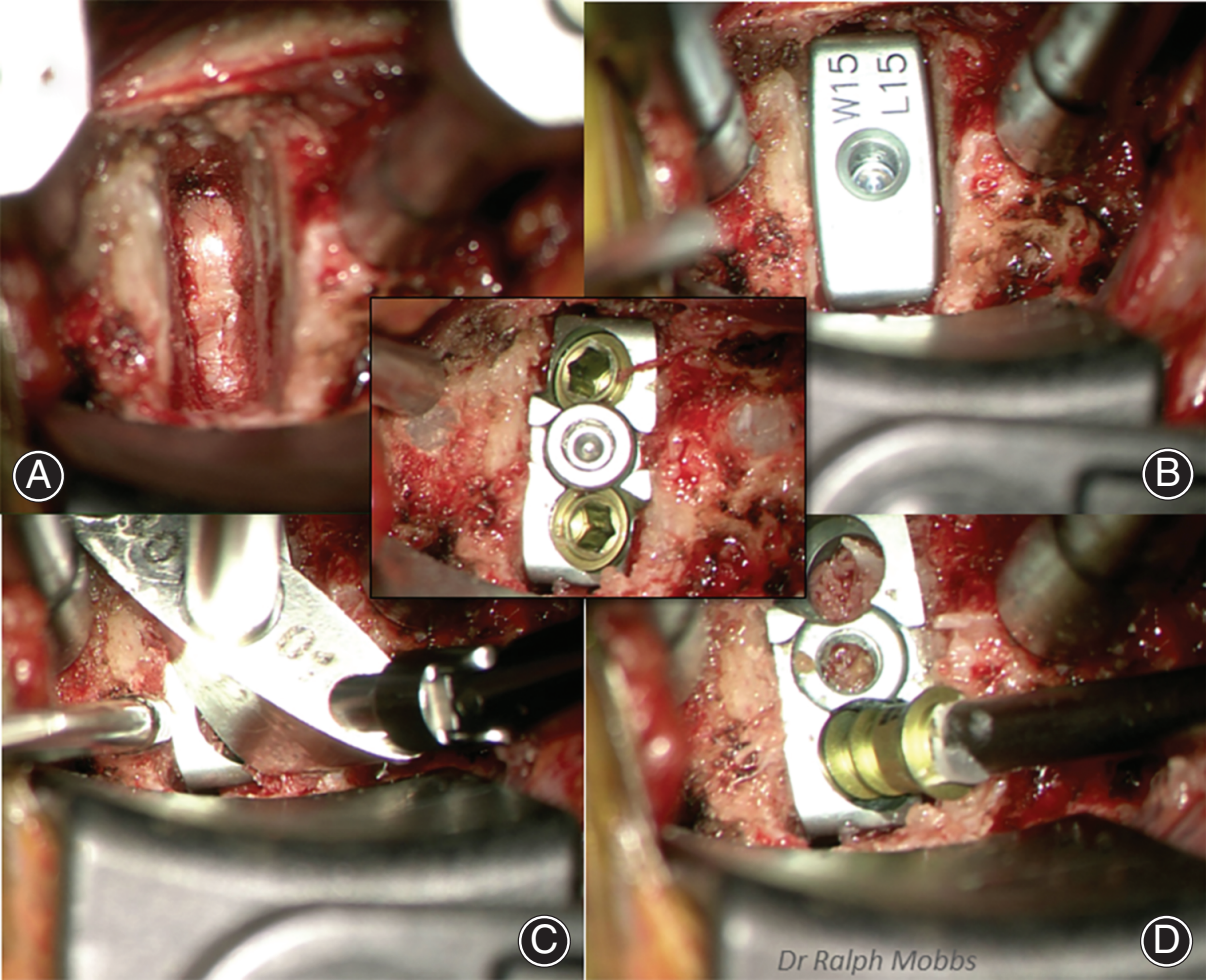
Sequence of anterior cervical discectomy and fusion (ACDF) using the ACDF device: (A) Exposure with Trim Line and Casper retractor with discectomy and decompression of the neurological elements. (B) Trial implant to determine width and height of final prosthesis. (C) Implantation of Redmond titanium (Ti)/polyetheretherketone (PEEK) cage with Awl guide on inserter. (D) ×2 screw integral fixation. (Inset) Final appearance.

In all cases, following implant impaction and verification on lateral X‐ray, integral fixation was applied *via* the use of ×2 self‐tapping screws (Fig. 8D). Prior to wound closure, intraoperative anteroposterior and lateral plain radiographs were obtained to confirm the correct implant and screw positioning. All patients were advised to wear a cervical orthosis postoperatively for a period of 2 weeks. Postoperative pain relief included a low dose of non‐steroidal anti‐inflammatory drug (NSAID) and paracetamol.

### *Outcome Measures*

#### *Radiographic Assessment*

Radiographic fusion was assessed by an independent radiology practice (Southern Radiology, Sydney, Australia) with no conflict of interest with regards to the study outcome. Anteroposterior and lateral cervical radiographs were performed at day 1 and week 6 postoperatively for radiographic assessment of the implant to confirm that there was no implant failure, delamination or movement from the original implantation position. Evidence of delamination of the titanium/PEEK interface was determined at each follow‐up time point^14^. Computed tomography (CT) scans were performed at 3 months to assess the early fusion status of bone growth through the implant and the absence of lucency, and at 6 months if unsure of fusion status. Fusion was considered successful if bridging bone incorporating the graft and adjoining endplates was apparent, with evidence of graft remodeling and new bone formation, restoration of interbody space, and no hardware/implant failure^16^.

Clinical outcomes were assessed preoperatively and 6 months postoperatively. Patients were asked to quantify neck and arm pain on a visual analogue scale (VAS) ranging from 0 (no pain/discomfort) to 10 (worst pain/discomfort imaginable) preoperatively and postoperatively. Functional outcome was measured using the neck Oswestry disability index (NODI). In addition, patients were assessed according to the Quality of Life 12‐Item Short Form (SF‐12) Health Survey, with their procedure determined using the patient satisfaction index (PSI) as described by Palit *et al*.^17^ at final follow‐up.

### *Statistical Analysis*

Descriptive data are represented as means ± SD (range, minimum–maximum). All datasets were tested for normality with the D’Agostino and Pearson omnibus normality test. Unpaired nonparametric data was analyzed using the Mann–Whitney *U* test and parametric data with an unpaired *t* test for comparison of the results. The Wilcoxon signed rank test was used for nonparametric, symmetrically distributed data and a paired *t* test was used for parametric data comparing preoperative and postoperative variables within patient groups. Statistical significance was set at *P* < 0.05. All analyses and graphs were generated using a commercial software package (GraphPad Prism version 5.01, GraphPad Software, USA).

## Results

### *Patient Demographics*

From 50 consecutive patients in the original dataset, 47 patients with 58 operative levels met the inclusion criteria. A total of 37 patients received a single level ACDF, 9 patients double level and 1 patient triple level. There were no patient deaths. There were 27 men and 20 women, with a mean age of 57 ± 14.5 years (range, 28–76 years) and a mean follow‐up period of 7.9 months (range, 6–10.8 months). Nine patients reported that they were smokers. There were 6 diabetics and 7 patients receiving workers’ compensation coverage for their surgery. Neck pain was present in almost all patients, with the main indication for surgery being cervical disc herniation associated with radiculopathy or spinal stenosis and cord compression. Within the non‐trauma cohort, the mean preoperative symptom length was 2.7 years (range, 9 weeks to 18 years). All patients were operated on between the surgical levels C_3_ and C_7_, with 10 multiple level ACDF procedures and 37 single level ACDF procedures.

### *Radiological Outcomes*

**Figure 9:**
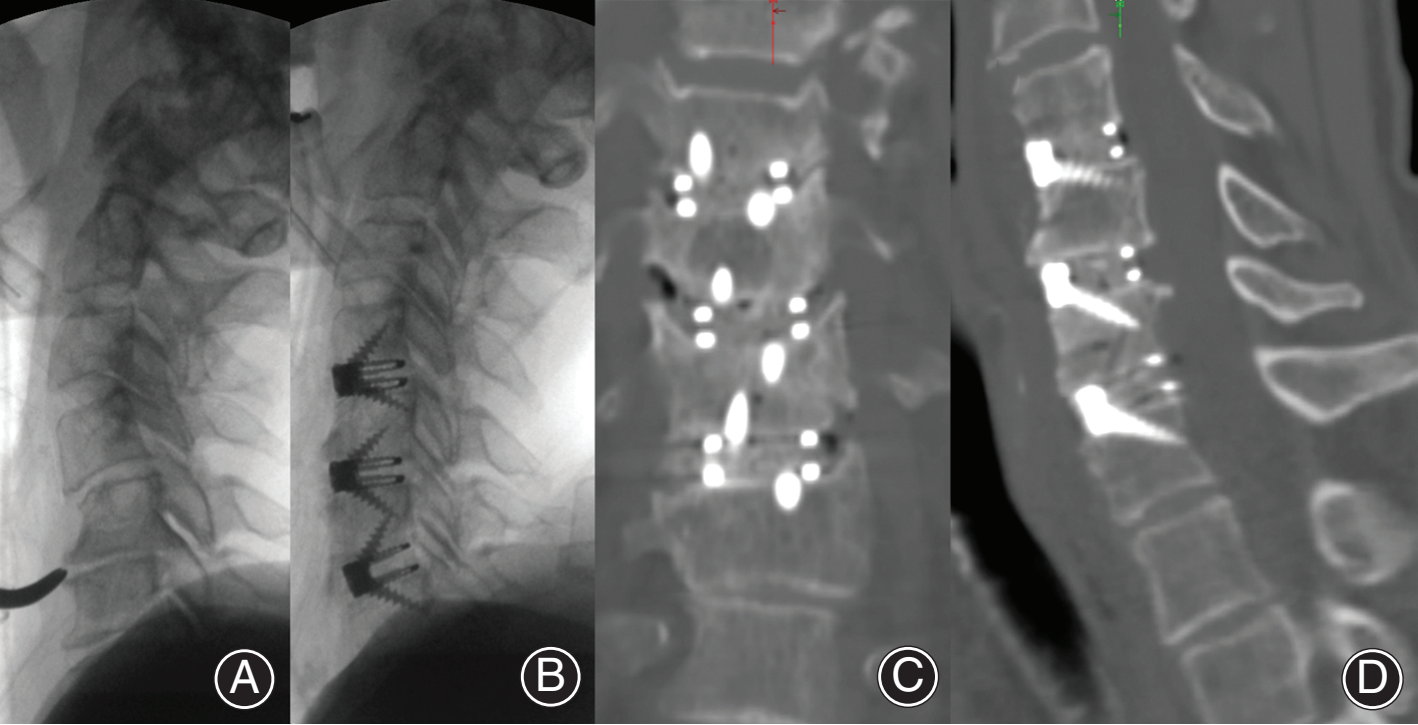
Fusion 3 months postoperatively. (A) Preoperative X‐ray. (B) Intraoperative X‐ray. (C, D) Difficult fusion environment with ×3 level anterior cervical discectomy and fusion (ACDF), with features of interbody fusion at all levels.

A fusion rate of 96% (45/47) was achieved, with evidence of bridging bone between endplates on CT scanning at 6 months postoperation. The presence of titanium endplates did not interfere with fusion assessments (Fig. 9). Evidence of graft remodeling at the interface with the endplate was noted.

### *Clinical Outcomes*

#### *Visual Analogue Scale Score*

Neck and arm pain scored using the VAS showed significant improvement (*P* < 0.001) between preoperative and postoperative groups. Overall pain improved from 7.3 ± 1.7 to 2.3 ± 1.5, with an average improvement of 5.0. These results are consistent with reported outcomes for ACDF surgery in the degenerative population^18^.

#### *SF‐12 Score*

Preoperative SF‐12 scores were physical component summary score (PCS) 37.8 ± 6, mental component summary score (MCS) 36.0 ± 7, with mean postoperative improvement of scores of 4.2 for PCS to 42.7 ± 8 and a mean postoperative improvement of 11.0 for MCS to 49.8 ± 8. This was statistically significant (*P* = 0.025) for MCS but not for PCS.

#### *Neck Oswestry Disability Index Score*

Significant mean neck ODI (NODI) scores preoperatively were 47.0 (SD ± 15.2), with a mean improvement of 22 (SD ± 7.8) (*P* = 0.04).

A summary of VAS, SF‐12 and NODI is provided in table 1.

**Table 1:** Patient clinical outcomes (mean ± standard deviation)

Evaluation criterion	Preoperative score	Postoperative score	Improvement
VAS	7.3 ± 1.7	2.3 ± 1.5	5.0
SF‐12			
PCS	37 ± 6	43 ± 8	4
MCS	36 ± 7	47 ± 8	11* (*P* = 0.025)
NODI	47 ± 15.2	22 ± 7.8	25* (*P* = 0.04)

* *P* < 0.05

MCS, mental component summary score; NODI, neck Oswestry disability index.; PCS, physical component summary score; SF‐12, Quality of Life 12‐item Short Form; VAS, visual analogue scale

#### *Odom's Assessment Results*

According to Odom's criteria, there were 39 excellent, 7 good, 3 fair, and no poor outcomes, with 92% of patients achieving a good–excellent clinical outcome. The 2 trauma patients were not included in the Odom's assessment, as noted above (Table 2).

**Table 2:** Clinical and radiological outcomes of Ti, PEEK, and Ti/PEEK cages^19^, ^20^, ^21^, ^22^, ^23^, ^24^, ^25^, ^26^, ^27^, ^28^, ^29^

Cage material	Good‐to‐excellent clinical outcome (%)	Fusion rate at 3 months (%)	Fusion rate at 6 months (%)	Fusion rate at 12 months (%)	Subsidence (%)
Titanium^20^, ^22^, ^27^, ^28^	46–95	—	37.2–97	86.5–99	13–45
PEEK^19^, ^20^, ^23^, ^24^, ^25^, ^26^, ^27^, ^28^, ^29^	74–100	—	61.1–96	93–100	5–15
Ti/PEEK (current study)	92	—	96	—	8.3 (4/48)

PEEK, polyetheretherketone; Ti, titanium.

### *Complications*

Complications were divided into approach‐related and implant‐related issues. There were no implant complications and there was no evidence of delamination in any cases. Additional attention was focused on 6‐month fine‐cut CT to determine the presence of implant failure at the Ti/PEEK junction, which was not present in any cases. Approach‐related complications included one case of recurrent laryngeal nerve palsy that had improved at the 4‐month follow‐up mark. There was 1 case of postoperative hematoma requiring an additional procedure for clot removal within 12 h of the index procedure. The patient proceeded to have an excellent clinical outcome at 6 months follow‐up.

## Discussion

A Cochrane systematic review concluded that interbody fusion techniques employing autograft yielded higher fusion rates than allograft and synthetic bone substitute techniques; however, donor site morbidity associated with autograft has fueled the growing interest in alternative materials^30^, ^31^. Furthermore, authors have questioned the current validity of the statement that “autograft is the gold standard”^32^. In this study, the combination of a Ti/PEEK cage, with integral fixation, with allograft, proved to be an effective and safe ACDF design and material combination, resulting in statistically significant improvements in pain and function at 6 months follow‐up.

### *Interbody Cage Material Properties*

Polyetheretherketone is a semicrystalline polyaromatic linear polymer and thermoplastic material of high molecular weight, which is biologically inert, radiolucent, and non‐resorbable^19^. For interbody spacers, PEEK provides a hard frame that can resist spinal loading, thereby providing initial stability while having an elastic modulus similar to that of bone to minimize graft subsidence^33^. Titanium can be modified to improve both ongrowth and ingrowth^34^, ^35^, ^36^. Ongrowth of bone is the direct apposition of bone to the surface. Ingrowth involves the interlocking or bone growth “into” the surface of a material, requiring a 3D structure with pores open to the outside. These modifications are aimed at influencing the way tissues integrate with the implant material^37^. The presence of titanium at the interface between the host and the device has the potential benefits of the hydrophilic and osteoconductive nature of titanium compared the hydrophobic nature of PEEK alone^38^. Titanium in the current device, however, is not a coating which minimizes the potential for delamination at the PEEK–titanium interface.

Previous studies have compared the efficacy of Ti and PEEK cages in both single and multi‐level ACDF. Studies have also shown PEEK to have better long‐term maintenance of clinical height, lower rates of subsidence, and improved clinical outcomes^39^. These advantages are attributed to PEEK's elastic modulus; however titanium implants have an exceptional ability to support direct bone ongrowth, and have a surface structure that is comparably resistant to microbial adhesion^40^, ^41^. By combining these materials in a composite cage, the advantages of both materials may theoretically be utilized. This study provides data for composite cages, with integral fixation, and demonstrates that they achieve comparable radiological and clinical outcomes to single material devices within a 6‐month follow‐up^19^, ^20^, ^21^, ^22^, ^23^, ^24^, ^25^, ^26^, ^27^, ^28^, ^29^.

### *Graft Choice*

Although the literature comparing implant materials and designs in ACDF is vast, information on the clinical impact of interbody graft choice is limited. Successful fusion is dependent on not only osteogenic potential and osteoinductive factors but also the structural scaffold that aids neovascularization and bony ingrowth^42^. Graft materials should promote osseointegration by providing an osteoconductive scaffold that can participate in the fusion. However, graft material inside a cage may also play an additional role in mechanical load distribution and reduce local stress concentrations^43^. Subsidence is thought to occur in relation to high pressures delivered through interbody spacers over a small surface area. As a result, PEEK cages, which have an elastic modulus closer to cancellous bone, experience lower rates of subsidence^39^. There is very little data comparing the rates of subsidence between patients with and without grafting, as well as graft types; however, it is known that autograft, allograft, and synthetic bone graft substitutes each have different mechanical and osteoconductive capacity. The mechanical properties of allograft bone are dependent on its sterilization treatment. While ionizing radiation sterilization of allograft increases brittleness and effects mechanical load bearing, SCCO_2_ treatment maintains the graft's intrinsic mechanical properties^44^, ^45^. All implants in our study were grafted with SCCO_2_ allograft, which was carefully packed into the cage and can be hypothesized to play a role in improved load distribution at the device–endplate interface and, therefore, prevention of subsidence; however, further studies are required to determine the role of this interbody graft choice.

### *Integral Fixation Design*

Integral fixation aims to reduce the amount of micro‐motion at the graft–host interface, graft settling, and kyphotic deformity, but also add to costs, risks, and operative time^46^. The role of integrated plate devices as provided by low‐profile designs including the Zero‐P (Synthes CmbH Switzerland, Oberdorf, Switzerland) and the ROI‐C cervical cage (LDR Holding Global Corporation, France) have been designed to assist surgeon work flow, and ease of implantation. By streamlining anterior plating into a stand‐alone device, these designs aim to minimize implant‐to‐soft tissue impact, reducing dysphagia rates and other plate‐related complications, while still reducing the risk of subsidence, pseudoarthrosis and cervical kyphosis^11^, ^47^. Early results have shown these designs to achieve good clinical outcomes, with lower levels of dysphagia and shorter operation times^47^, ^48^. The current study combines the positive benefits of both integral fixation technology with the dual material combination of titanium and PEEK.

### *Study Limitations*

A primary limitation of this study is the relatively small numbers involved. Incomplete follow‐up data on patients was a difficulty in our study, as is common for clinical studies; in our particular cohort, this was primarily related to patients from rural areas being unable to access medical imaging centers in the required timeframe of the study.

The assessment of interbody fusion and the integration of the Ti endplate remains a challenge. As there are no universally accepted criteria for determining radiological fusion, it is often difficult to arrive at a true assessment of fusion based on plain radiography alone, particularly when synthetic cages are used. Our study utilized fine‐cut CT scans with reconstructions, which has been shown to be more reliable and sensitive for the detection of pseudoarthrosis than plain radiography^49^, ^50^. In addition, it was noted that the Ti‐endplates did not interfere with fusion assessments on either radiographs or CT.

### *Conclusion*

In this study, we have found that using an integral fixation Ti/PEEK interbody cage containing allograft in anterior cervical discectomy and fusion was a safe and effective treatment for degenerative and traumatic cervical pathologies. Enhancement of PEEK cages with titanium endplates is likely to assist with early integration of the prosthesis with the surrounding bone and vertebral endplate. Further studies are required to determine if the usage of a composite design improves implant longevity by limiting subsidence as well as stress shielding and associated complications.
